# Perceptions and knowledge of telemedicine in Ecuadorian practicing physicians: an instrument adaptation, validation and translation from English to Spanish

**DOI:** 10.1186/s12889-021-11826-1

**Published:** 2021-10-02

**Authors:** Geovanny Alvarado-Villa, Christian KuonYeng-Escalante, Nicolás Sagñay-Pinilla, Carlos Vera Paz, Ivan Cherrez-Ojeda

**Affiliations:** Universidad Espíritu Santo, Samborondón, Ecuador

**Keywords:** Telemedicine, Healthcare, Questionnaire, Perceptions

## Abstract

**Background:**

During the COVID-19 pandemic, multiple countries have taken measures, such as isolation and quarantine, to prevent person-to-person spread of disease. These actions forced many physicians to adopt new techniques, such as telemedicine, to continue patient care, which has proven to be useful in continued care for those with non-COVID-19 pathologies. Various factors, such as security, confidentiality, cost-effectiveness, comfort, and the risk of malpractice, influence the perception of telemedicine among medical practitioners. The aim of this study was to adapt an existing instrument and validate it into a new Spanish version. The instrument is about the perceptions and knowledge of telemedicine in healthcare professionals.

**Methods:**

The original questionnaire surveyed 6 domains with 40 questions, and each question was measured with a five-point Likert scale ranging from very high [5] to very low [1]. The survey was translated to Spanish using machine translation. The translation was reviewed independently, and then, a consensus was achieved regarding minor changes in the syntax of the survey to facilitate understanding. After expert feedback and questionnaire review, the research team members proposed reducing the instrument to 13 items in 4 domains due to the similarity of some questions. The sample was divided into 2randomly selected groups. Eligibility criteria included physicians providing private or public services with active medical/clinical practice.

**Results:**

In total, 382 surveys were collected and separated into two random samples, S1 and S2 (198 and 184, respectively). In exploratory factor analysis (EFA), the 13 items were grouped into four theoretical domains, and item 7 presented cross loading between factors and was removed. Confirmatory factor analysis was performed to assess the scale reliability and interscale associations; three models were tested. Global Cronbach’s alpha for internal consistency was 0.76 for the EFA. The goodness of fit measures root mean square error of approximation and comparative fit index were 0.009 and 0.999, respectively, for the best model.

**Conclusions:**

The translated instrument was clear, with adequate internal consistency, readability, and appropriate for application in the physician setting. This validated questionnaire made it possible to evaluate physicians’ knowledge of telemedicine to increase its use, especially during the COVID-19 pandemic.

**Supplementary Information:**

The online version contains supplementary material available at 10.1186/s12889-021-11826-1.

## Background

The use of information and communication technologies (ICTs) in healthcare has significantly changed the physician-patient relationship [1]. Telemedicine is the implementation of ICTs to deliver distant medical care [2]. During the COVID-19 pandemic, multiple countries took public health measures. Isolation and quarantine were community containment plans to prevent person-to-person spread of disease [3]. This unique situation forced many physicians to adopt new techniques to continue patient care [4]. Telemedicine has proven to be a useful tool during the pandemic. As a result, it has been used in the assessment and triage of patients with COVID-19 to reduce healthcare load. Nevertheless, it has proven to be useful for the continued care of those with non-COVID-19 pathologies [5–7]. In addition, telemedicine may reduce costs due to decreased prolonged hospital stays [8].

Adaptation to new technologies is generally a process of constant cost-benefit evaluation [9]. For instance, various factors, such as security, confidentiality, cost-effectiveness, comfort, and the risk of malpractice, influence the perception of medical practitioners [10]. Kuo et al. (2015) described several factors, such as attitude, subjective norms, and perceived behavioral control, that could affect the intention of physicians to adopt new technologies [11].

There are some validated English instruments for evaluating physicians’ perceptions that were not selected. These questionnaires focus on specialized subjects regarding perceptions and knowledge of telemedicine, such as telerehabilitation [12, 13], and they are designed for specific medical specialties. Ayatollahi et al. [10] talks about the perceptions and knowledge of telemedicine in general terms, this is the reason why it was selected by the investigators for the adaptation into Spanish and generate a new instrument.

The appropriate assessment of clinician knowledge and perceptions of telemedicine is essential. Therefore, this study created a reliable tool that can detect changes in physicians’ perceptions of the use of telemedicine in the future [14, 15]. The aim of this study was to adapt an existing instrument and validate it into a new Spanish version. The instrument is about the perceptions and knowledge of telemedicine in healthcare professionals.

## Methods

### Study design

All methods were carried out in accordance with relevant guidelines and regulations. The study was conducted in Ecuadorian physicians, for 6 weeks in a period between march 23rd to may 11th of 2020. Surveys collected were recorded in a standardized database for process and statistical analysis. Data security and protection was preserved at all points during the process. Eligibility criteria included physicians providing private or public services with active medical/clinical practice. The eligibility criteria excluded non-Ecuadorian physicians, because the aim was to adapt a questionnaire based on the region. The sample size was determined by multiplying the number of questions (13 items) by 5 (lower limit) and 20 (upper limit), yielding a needed sample size of 65 to 250 doctors, as proposed by Suhr (2005) and other validation studies in an Ecuadorian population [16–19]. The population for this investigation was selected by a nondiscriminative selective snowball sampling method, because of social distancing due to COVID-19 pandemic. The instrument was provided on webinars and allowed the recruitment of 404 physicians.

The original questionnaire included 6 domains with 40 questions, and each question was measured with a five-point Likert scale ranging from very high [5] to very low [1] [10]. The authors do not consider demographic characteristics as a domain because they do not measure a construct. Yang et al. (2018) proposed a model for the adoption of machine translations (MTs) [20]. Consequently, the survey was translated into Spanish using MT. The translation was reviewed independently by each research team member, and then, a consensus was achieved regarding minor changes needed in the syntax of the survey to facilitate understanding.

The translated instrument was converted into a Google Form survey and was implemented as a pilot test among 65 physicians. The survey objective was to ensure that the vocabulary, understanding, design, and time needed to complete the survey were appropriate enough to ensure stability and comprehension [21].

After the expert feedback and questionnaire indicators that resulted in the pilot test, the research team members proposed reducing the instrument to 13 items in 4 domains. Three domains were modified or excluded: “Clinicians’ perception of the advantages of telemedicine technology”, “Clinicians’ perception of the telemedicine technology ease of use” and “Clinicians’ perception of the necessity of telemedicine technology”. The former was modified because physicians do not receive training on the use of ICTs and the similarity between some questions of this domains and some questions of the remain domains. The second one was modified and changed to “Perception of the utility of telemedicine”. In the same way, questions that could not be used due to differences in administrative characteristics (budget management, conferences, training or guidelines) were excluded. From the original instrument “Clinicians’ perception of the advantages of telemedicine technology” and “Clinicians’ perception of the necessity of telemedicine technology” were excluded.

The questionnaire was completed by 404 participants, and 22 of them were excluded because they were not Ecuadorian. The sample was divided into 2 randomly selected groups. The first group consisted of 198 doctors for sample 1 (S1) to perform exploratory factor analysis (EFA). The second group included 184 doctors for sample 2 (S2) for confirmatory factor analysis (CFA).

### Statistical analysis

#### Descriptive analyze

A descriptive statistical analysis was implemented for sociodemographic data. Qualitative variables are presented as frequencies and percentages, and quantitative variables are presented as the means and standard deviations. Each item was measured using its mean response value. Age, sex, educational level, and workplace were used as independent variables in each analysis. They were examined using skewness and kurtosis for normality. Statistical significance was considered as a *p* < 0.05. All statistical analyses were performed using SPSS for Windows (version 25.0; SPSS Inc., Chicago, Illinois).

#### Psychometric analysis

The Kaiser-Meyer-Olkin (KMO) measure was calculated to evaluate the sampling adequacy of each item on the anti-image of the correlation matrix [22]. All sampling measure cutoff points were set at 0.5 [22]. Bartlett’s test of sphericity indicated that the analysis was suitable for the scale [23]. Commonality analysis was performed, and after this procedure, the questionnaire was reduced to ensure construct parsimony and usability.

The scale structure was determined by several indicators. Initially, eigenvalues were extracted. A factor structure with eigenvalues above 1 was selected as the criterion for EFA. Maximum likelihood estimation (MLE) was performed and supported the reduction of the instrument to 4 domains: “Knowledge about telemedicine”, “Perception of the utility of telemedicine”, “Perception of the disadvantages of telemedicine”, and “Knowledge of the security of telemedicine”. The chi square showed a nonsignificant result, supporting the MLE.

The internal consistency was calculated by Cronbach’s alpha for both the full scale and for each of the subscales found in the EFA. Cronbach’s alpha measures the interrelation of the items, with values ranging between 0 and 1 [24]. The results above the threshold of > 0.7 were considered acceptable. A value > 0.9 suggests redundancies, indicating that the instrument should be shortened [25].

Other tests were used to indicate goodness of fit: Chi square, root mean square error of approximation (RMSEA: range 0–1, with a recommended result ≤0.06), normed fit index (NFI: range 0–1, with a recommended result > 0.90), Tucker Lewis index (TLI: 0–1, with a recommended result > 0.90), comparative fit index (CFI: range 0–1, with a recommended result > 0.90), parsimony ratio (range 0–1, with a recommended result of approximately 1), parsimony NFI (PNFI: range 0–1, with a recommended result > 0.50), and parsimony CFI (PCFI: range 0–1, with a recommended result > 0.50) [26, 27].

#### Readability

The Fernandez-Huerta index and Crawford index were used to assess readability and estimate grade level, respectively. Scores were reported in a range of 0–100, with higher results indicating greater readability, and a result of 60–70 represented easy understanding for the population of ~ 15 years old [28].

## Results

With a response rate of 95%, 382 participants completed the survey. Two hundred twenty-four of the participants were male (58.6%). Most of the participants had a permanent job (88,7%). Approximately 66.8% of the physicians were medical specialists, and 22.3% are currently working as primary care physicians. Their workplace was separated in almost equal percentages: hospital with 23%, clinic 24.3%, both hospital and clinic with 23.6%, private practice with 20.9% and others with 8.1%. The 382 surveys were collected and separated into two random samples, S1 and S2 (198 and 184, respectively). EFA and CFA was performed in S1 and S2, respectively (see Table 1).

**Table 1:** Sociodemographic characteristics of the population

Variables		S1		S2	
		Frequency (n)	Percentage (%)	Frequency (n)	Percentage (%)
Sex	Male	115	58.1	109	59.2
	Female	83	41.9	75	40.8
Employment type	Permanent	174	87.9	165	89.7
	Temporary	24	12.1	19	10.3
Education level	General doctor	42	21.2	46	25
	Medical specialist	132	66.7	123	66.8
	Master’s degree	13	6.6	7	3.8
	PhD	11	5.6	8	4.3
Work experience (years)	1—5	19	9.6	16	8.7
	6—10	21	10.6	19	10.3
	11—15	23	11.6	30	16.3
	16—20	25	12.6	25	13.6
	Over 20	110	55.6	94	51.1
Workplace	Hospital	60	30.3	42	22.8
	Clinic	47	23.7	46	25
	Both	36	18.2	38	20.7
	Other	55	27.8	58	31.5

### Descriptive

Individual items were expected to be non-normal. Consistent with this, the skewness ranged from − 1.13 (item 13) to 0.28 (item 2). The kurtosis ranged from − 1.03 (item 8) to 0.84 (item 13). Individual items also had median values ranging from 1.02 (item 13) to 1.28 (item 8). The clinicians’ knowledge about telemedicine is limited, according to item 1, 33% (average) and 25.4% (low), item 2, 30.1% (average) and 30.1% (low) and item 3, 32.7% (average) and 27.7% (low). The perceptions of the utility of telemedicine was average, according to item 4, 32.5% (high) and 28.5% (average), item 5, 35.6% (average) and 23.3% (high) and item 6, 343% (average) and 23.8% (very low) (see Table 2).

**Table 2:** Descriptive statistics of the items of the proposed instrument

Item		1	2	3	4	5	Mean	SD	Median	Skewness	Kurtosis
		Very low	Low	Average	High	Very high					
1	Frequency (n)	81	97	126	62	16	2.57	3	1.12	0.17	−0.76
	Percentage (%)	21.2%	25.4%	33.0%	16.2%	4.2%					
2	Frequency (n)	86	115	115	55	11	2.45	2	1.08	0.28	−0.70
	Percentage (%)	22.5%	30.1%	30.1%	14.4%	2.9%					
3	Frequency (n)	88	106	125	51	12	2.46	2	1.08	0.25	−0.67
	Percentage (%)	23.0%	27.7%	32.7%	13.4%	3.1%					
4	Frequency (n)	48	67	109	124	34	3.08	3	1.16	−0.29	−0.80
	Percentage (%)	12.6%	17.5%	28.5%	32.5%	8.9%					
5	Frequency (n)	62	64	136	89	31	2.90	3	1.17	−0.12	−0.77
	Percentage (%)	16.2%	16.8%	35.6%	23.3%	8.1%					
6	Frequency (n)	91	90	131	55	15	2.51	3	1.12	0.19	−0.76
	Percentage (%)	23.8%	23.6%	34.3%	14.4%	3.9%					
7	Frequency (n)	34	51	120	122	55	3.30	3	1.14	−0.37	−0.53
	Percentage (%)	8.9%	13.4%	31.4%	31.9%	14.4%					
8	Frequency (n)	94	93	89	71	35	2.63	3	1.28	0.27	−1.03
	Percentage (%)	24.6%	24.3%	23.3%	18.6%	9.2%					
9	Frequency (n)	53	83	106	93	47	2.99	3	1.23	−0.04	−0.95
	Percentage (%)	13.9%	21.7%	27.7%	24.3%	12.3%					
10	Frequency (n)	44	68	99	103	68	3.22	3	1.26	−0.22	−0.95
	Percentage (%)	11.5%	17.8%	25.9%	27.0%	17.8%					
11	Frequency (n)	10	27	58	105	182	4.10	4	1.07	−1.08	0.40
	Percentage (%)	2.6%	7.1%	15.2%	27.5%	47.6%					
12	Frequency (n)	9	24	58	107	184	4.13	4	1.04	−1.11	0.54
	Percentage (%)	2.4%	6.3%	15.2%	28.0%	48.2%					
13	Frequency (n)	11	19	59	128	165	4.09	4	1.02	−1.13	0.84
	Percentage (%)	2.9%	5.0%	15.4%	33.5%	43.2%					

### Exploratory factor analysis

The Cronbach’s alpha for the pilot test was 0.94. In the EFA, the 13 items were grouped into four theoretical domains, and item 7 “To what extent does ease of use of telemedicine technology facilitate its learning?” presented cross loading between factors and was removed. However, item removal at this stage did not improve the reliability statistics (see Table 3). The global Cronbach’s alpha for internal consistency was 0.76.

**Table 3:** Factor Analysis Pattern Matrix

Questions	Domains			
	1	2	3	4
1	0.910			
2	0.983			
3	0.824			
4			0.786	
5			0.825	
6			0.790	−0.380
7		0.476	0.601	
8				0.774
9				0.741
10				0.782
11		0.922		
12		0.945		
13		0.927		

### Confirmatory factor analysis

**Fig. 1:**
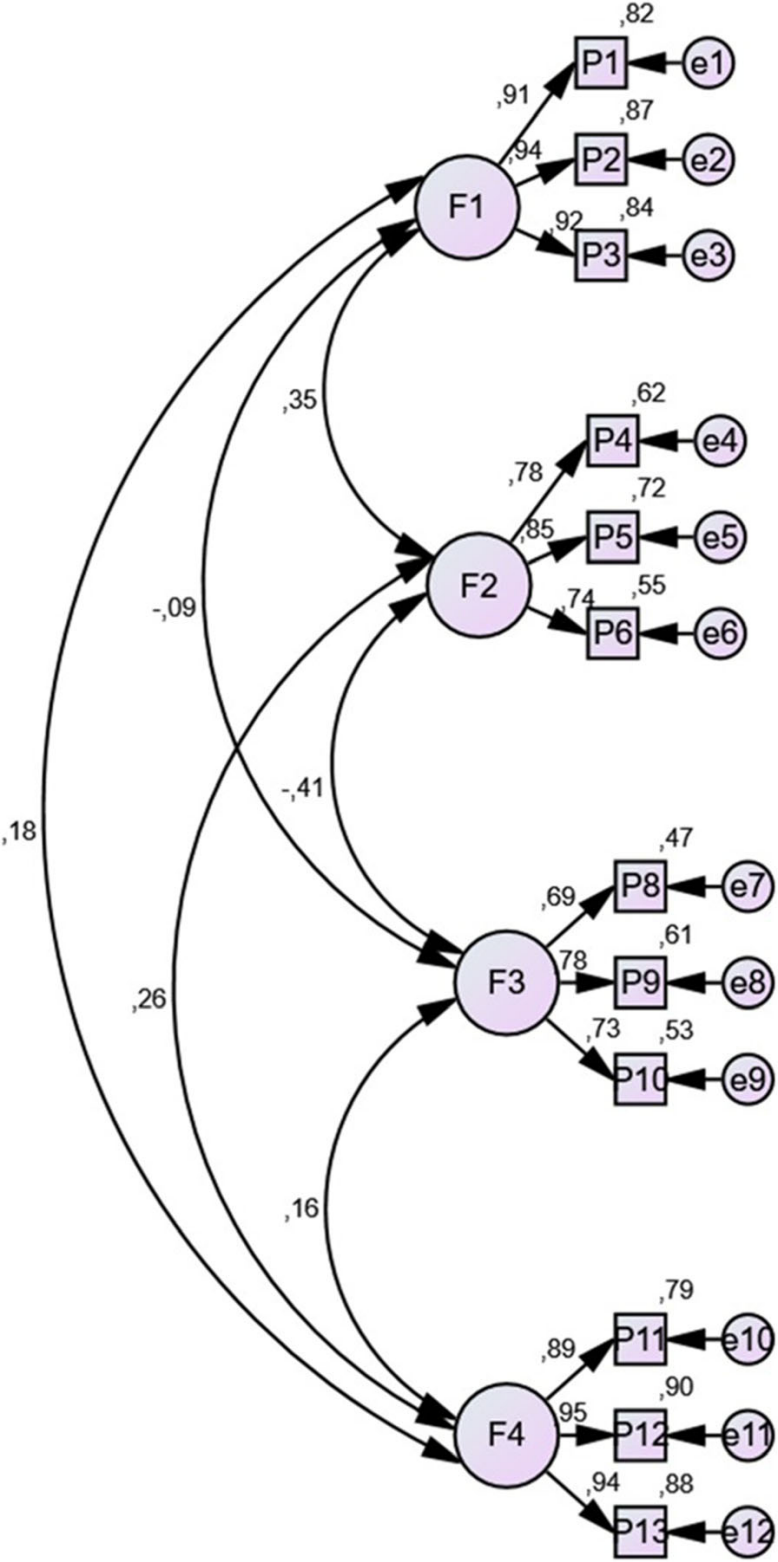
Model 3- Unifactorial exclusion of the seventh item

CFA was performed to assess scale reliability and interscale associations. Three models (M1, M2, M3) were tested to determine the best reliability (see Tables 4 and 5). The removal of item 7 at this stage provided the best goodness of fit measures for the final model. Goodness of fit measures are shown in Table 5 (see Table 6). Figure 1 shows the results for model 3 with RMSEA and CFI values of 0.009 and CFI 0.999, respectively (see Fig. 1).

**Table 4:** Comparison of Cronbach’s alphas of the three models, with Model 3 excluding item 7

Sample 1			Sample 2	
Questions	Cronbach’s Alpha per domain-Model 1	Total Cronbach’s Alpha-Model 1	Cronbach’s Alpha per domain-Model 2	Total Cronbach’s Alpha-Model 2
1	0.93	0.76	0.942	0.742
2				
3				
4	0.834		0.803	
5				
6				
7				
8	0.807		0.772	
9				
10				
11	0.952		0.947	
12				
13

**Table 5:** Cronbach’s alpha of model 3

Domains	Questions	Cronbach’s Alfa per domains-Model 3	Total Cronbach’s Alfa-Model 3
Knowledge about telemedicine	1. ¿En qué medida está familiarizado con la telemedicina?	0.942	0.715
	*To what extent are you familiar with telemedicine technology?*		
	2. ¿En qué medida está familiarizado con las aplicaciones médicas de la telemedicina?		
	*To what extent are you familiar with the medical applications of telemedicine technology?*		
	3. ¿En qué medida está familiarizado con las herramientas de la telemedicina?		
	*To what extent are you familiar with telemedicine tools?*		
Perception of the utility of telemedicine	4. En su opinión, ¿En qué medida la telemedicina es efectiva para reducir los costos de la atención al paciente en los hospitales?	0.833	
	*In your opinion, to what extent is telemedicine effective in reducing the costs of patient care in hospitals?*		
	5. En su opinión, ¿En qué medida la tecnología de telemedicina ahorra tiempo a los médicos?		
	*In your opinion, to what extent does telemedicine technology save clinicians’ time?*		
	6. En su opinión, ¿En qué medida la tecnología de telemedicina proporciona una atención médica mejor y más rápida?		
	*In your opinion, to what extent does telemedicine technology provide faster and better medical care?*		
Perception of the disadvantages of telemedicine	7. En su opinión, ¿En qué medida la tecnología de telemedicina pone en peligro la privacidad del paciente?	0.772	
	*In your opinion, to what extent does telemedicine technology endanger patient privacy?*		
	8. En su opinión, ¿En qué medida la tecnología de telemedicina reduce la eficiencia de la atención al paciente?		
	*In your opinion, to what extent does telemedicine technology reduce the efficiency of patient care?*		
	9. En su opinión, ¿En qué medida la tecnología de telemedicina aumenta la mala práctica médica?		
	*In your opinion, to what extent may telemedicine technology increase malpractice in healthcare?*		
Knowledge of the security of telemedicine	10. ¿En qué medida se debe crear un marco para evitar la violación de la confidencialidad de los datos cuando se utiliza la telemedicina?	0.947	
	*To what extent should a framework be created to prevent breaching data confidentiality when using telemedicine?*		
	11. ¿En qué medida la tecnología de telemedicina requiere una aclaración legal para los pacientes?		
	To what extent does telemedicine technology require legal clarification for patients?		
	12. ¿En qué medida la tecnología de telemedicina requiere un marco formulado y claro para acceder a la información médica?		
	*To what extent does telemedicine technology require a formulated and clear framework for access to medical information?*

**Table 6:** Goodness of fit measures for Model 1, Model 2 and Model 3

	Absolute adjustment measures		Incremental adjustment measures			Parsimony adjustment measures		
Models	Chi Square	RMSEA (IC)	NFI	TLI	CFI	PRATIO	PNFI	PCFI
M1	0.002	0.057 (0.034–0.078)	0.939	0.970	0.976	0.782	0.735	0.763
M2	0.176	0.032 (0.000–0.060)	0.961	0.992	0.994	0.758	0.728	0.753
M3	0.443	0.009 (0.000–0.049)	0.968	0.999	0.999	0.727	0.704	0.727

### Readability

A readability analysis was conducted. The results showed a Fernandez-Huerta score of 52.6 and a Crawford grade level of 6.6 [29]. These results indicated that these items were likely to be understandable by a typical 14- to 15-year-old individual and required a minimum of 6 years of schooling.

## Discussion

The results of this validation study achieved a parsimonious and reliable instrument. The results can be used to rapidly assess perceptions of new technology using data derived from a large cohort of medical practitioners. Several studies have concluded that the length of questionnaires has an inverse relation with the response rate [30, 31]. Due to the time constraints of physicians, the questionnaire was designed to be short and feasible.

Telemedicine has the potential to improve access to healthcare, especially in rural areas [32]. Regardless of its benefits, telemedicine has not been widely adopted. Readiness at various levels was the main factor determining the decision of health centers to not use telehealth “yet”. Some centers stated that there were other priorities, such as building patient portals, and others were exploring the process of implementing telehealth [32]. Some patients have negative beliefs about telemedicine, with the main belief being that telemedicine can lead to medical errors [33]. Knowledge of telemedicine is an important key to identifying negative issues. Consequently, healthcare professionals need to learn and adapt their communication and technology for better results.

Evidence from the scientific literature shows that MT can be very useful for developing reliable instruments [34]. The syntax of the target questions was simple enough for rapid translation into a different language with acceptable results. In addition, participant characteristics and sample size ensure that this instrument was appropriate for validation. Another strength of the validation was the heterogeneity of the sample, with physicians with various medical specialties responding to the questionnaire.

The goodness of fit measures for model 3 were better than those for models 1 and 2. Our instrument had readability and validity according to the CFA. In contrast, the original instrument had only correlations between items.

A positive relationship between domains 1 and 2 was shown in the final model due to the positive selection in domain 2 but negative selection in domain 3. Likewise, there was a weaker positive relationship between domain 1 and domain 4 and a negative relationship of domain 3 with domains 1 and 2.

There were some limitations to our study that need to be discussed. First, our population included only Ecuadorian physicians, and the sample may not be representative of other Hispanic countries. The original questionnaire this study derives its initial question set from does not possess a robust theoretical background for feature and domain selection. However, the concise methodology used allows for an adequate baseline to create a Spanish instrument. The study process included the removal of items to offer a simpler construct to derive a solid theoretical background from. The performance of the model suggests that the features selected represent to an adequate degree the problems addressed by the questionnaire. In the traditional questionnaire validation process, backwards translation is used for verification without a doubt that the original translation was valid. Considering that we used a machine learning model to perform the translation, its validity can be verified by switching the input and output. Therefore, the process of backwards translation was not formally performed in this study.

The original survey compiled 6 characteristic domains of the population and was validated using face and content validity methods. The original questionnaire had a Cronbach’s alpha coefficient of 0.73 [9]. Additionally, our instrument exhibited an acceptable KMO and a significant Bartlett and explained the variance of the 4 dimensions with a strong significant association. Furthermore, our questionnaire, unlike the original questionnaire, was tested using chi square, RMSEA, TLI, NFI, PNFI, CFI, and PCFI, indicating goodness of fit.

Although the use of ICTs among clinicians is high [35], according to the results of our study, clinicians’ knowledge about telemedicine is limited. Still with these results about knowledge of telemedicine, clinicians perceived that it is necessarily the use of it. The security of telemedicine is very important, and most clinicians agree that a framework should be created to prevent breaching data confidentiality. Additionally, the requirement of a formulated and clear framework for access to medical information and legal clarification for patients is needed. Previous studies showed similar results. Ashfaq et al. and Ayatollahi et al. concluded that clinicians’ knowledge was also low, and the majority of them thought that the usage of telemedicine was necessary [10, 36].

## Conclusions

Our study developed a translated and validated questionnaire to evaluate physician knowledge and perceptions of telemedicine. The translated instrument was clear, with adequate internal consistency, readability, and appropriate application in physician populations. We identified 12 items grouped into the four theoretical domains.

Our validated questionnaire could help to evaluate physicians’ knowledge of telemedicine especially during the COVID-19 pandemic and thereby contribute to the development of tools that can increase the use of telemedicine technologies. We identified the perceptions and knowledge of Ecuadorian physician regarding Telemedicine and recommend that these should be validated in other Hispanic populations.

## Supplementary Information

**Additional file 1.**

## Data Availability

The datasets used and analyzed during the current study are available from the corresponding author on reasonable request.

## Abbreviations

CEISH: Comité de ética e Investigación en Seres Humanos
CFA: Confirmatory factor analysis
CFI: Comparative fit index
EFA: Exploratory factor analysis
ICTs: Information and communication technologies
KMO: Kaiser-Meyer-Olkin
MLE: Maximum likelihood estimation
MT: Machine translation
NFI: Normed fit index
PCFI: Parsimony comparative fit index
PNFI: Parsimony normed fit index
RMSEA: Root mean square error of approximation
TLI: Tucker Lewis index
